# Supplementary material for: Machine learning and glioma imaging biomarkers
Source: Clin Radiol. Author manuscript; Available in PMC 2020 Jan 1. (PMC6927796; doi:10.1016/j.crad.2019.07.001)
Supplement: Supplementary Box 1 [file EMS84634-supplement-Supplementary_Box_1.docx]

Supplementary Box 1. Tools useful for ML and neuro-oncology applications.

| Tools such as scikit-learn, h20.ai, LIBSVM and WEKA are appropriate for classic ML models (such as for Random Forest and SVM). Both niftynet and DLTK are useful for deep learning when applied to medical imaging, and both build on the general purpose Tensorflow package. Other general purpose deep learning packages (not medical specific), such as PyTorch and MXnet are also available. All these packages (listed below) are open source with highly permissive licenses (normally Apache 2.0).  https://scikit-learn.org/stable/  https://www.h2o.ai/  https://www.csie.ntu.edu.tw/~cjlin/libsvm/  http://www.niftynet.io/  https://www.cs.waikato.ac.nz/~ml/weka/  https://dltk.github.io/  https://www.tensorflow.org/  https://pytorch.org/  https://mxnet.apache.org/ |
| --- |
