# Supplementary figures and images for: Machine learning and glioma imaging biomarkers
Source: Clin Radiol. Author manuscript; Available in PMC 2020 Jan 1. (PMC6927796; doi:10.1016/j.crad.2019.07.001)

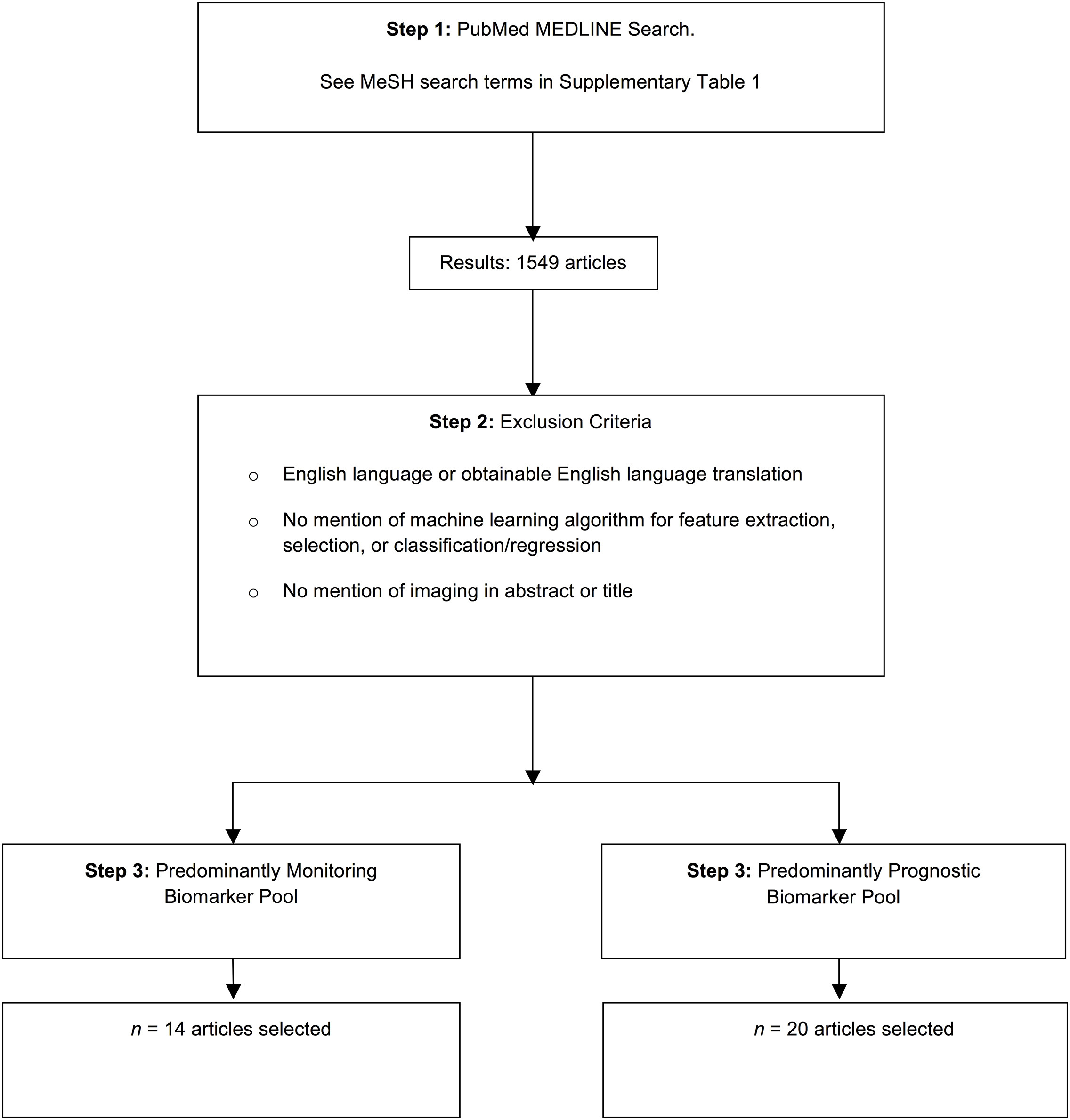

Supplement: Supplementary Figure 1 Literature Search Flow Diagram [file EMS84634-supplement-Supplementary_Figure_1_Literature_Search_Flow_Diagram.jpg]
