# Supplementary material for: Machine learning and glioma imaging biomarkers
Source: Clin Radiol. Author manuscript; Available in PMC 2020 Jan 1. (PMC6927796; doi:10.1016/j.crad.2019.07.001)
Supplement: Supplementary Table 1 [file EMS84634-supplement-Supplementary_Table_1.docx]

**Supplementary Table 1.** MeSH terms used for Search Strategy.

| **Terms** | **Filters** |
| --- | --- |
| glioblastoma[Title] pseudoprogression[Title] | Review, Last 10 years |
| glioblastoma[Title] machine learning[Title] |  |
| glioblastoma[Title] deep learning[Title] |  |
| "glioblastoma"[Title] "prediction"[Title] |  |
| "glioblastoma"[Title] "prognosis"[Title] |  |
| (high grade glioma[Title/Abstract]) AND pseudoprogression[Title/Abstract] |  |
| (high grade glioma[Title/Abstract]) AND (machine learning[Title/Abstract]) |  |
| (high grade glioma[Title/Abstract]) AND (machine learning[Title/Abstract]) |  |
| (high grade glioma[Title/Abstract]) AND prediction[Title/Abstract] |  |
| (high grade glioma[Title/Abstract]) AND prognosis[Title/Abstract] |  |
| (glioblastoma[Title/Abstract]) AND prediction[Title/Abstract] |  |
| (glioblastoma[Title/Abstract]) AND radiomics[Title/Abstract] |  |
|  |  |
| (glioblastoma[Title/Abstract]) AND radiomics[Title/Abstract] | Last 5 years |
| glioblastoma[Title/Abstract] AND pseudoprogression[Title/Abstract] |  |
| (glioblastoma[Title/Abstract]) AND machine learning[Title/Abstract] |  |
| (glioblastoma[Title/Abstract]) AND prediction[Title/Abstract] |  |
| (glioblastoma[Title/Abstract]) AND deep learning[Title/Abstract] |  |
| (glioblastoma[Title/Abstract]) AND prognosis[Title/Abstract] AND MRI[Title/Abstract] |  |
